# Wearable Technologies and Stress: Toward an Ethically Grounded Approach

**DOI:** 10.3390/ijerph20186737

**Published:** 2023-09-11

**Authors:** Stefano Canali, Beatrice De Marchi, Andrea Aliverti

**Affiliations:** Department of Electronics, Information and Bioengineering, Politecnico di Milano, 20133 Milano, Italy

**Keywords:** digital health, wearables, stress, ethics, philosophy of technology

## Abstract

The widespread use of digital technologies that can be worn on our bodies—wearables—is presented as a turning point for various areas of biomedical research and healthcare, such as stress. The ability to constantly measure these parameters, the perceived quality of measurement, and their individual and personal level frame wearable technology as a possibly crucial step in the direction of a more accurate and objective definition and measurement of stress for clinical, research, and personal purposes. In this paper, we discuss the hypothesis that the use of wearables for stress is also beneficial from an ethical viewpoint. We start by situating wearables in the context of existing methods and limitations of stress research. On this basis, we discuss the ethics of wearables for stress by applying ethical principles from bioethics (beneficence, non-maleficence, autonomy, justice), which allows us to identify ethical benefits as well as challenges in this context. As a result, we develop a more balanced view of the ethics of wearables for stress, which we use to present recommendations and indications with a focus on certification, accessibility, and inclusion. This article is, thus, a contribution towards ethically grounded wearable and digital health technology for stress.

## 1. Introduction

According to the American Institute of Stress [1], stress affects one-third of the global population, with significant connections with and implications on several health problems (e.g., mental health; cardiovascular diseases; eating disorders; gastrointestinal problems; and beyond) [2,3]. Despite its impact, several conceptual and methodological dimensions of stress as a biomedical phenomenon remain unclear and unsettled. Stress effects change between acute and chronic phases and the response to this event is expressed in very different ways among different individual people. This makes it difficult to provide a standard definition and identification procedure. As a result, currently, there is no commonly accepted definition of stress, which creates an uncertain basis for building standard stress level assessment and measurement methods [4]. The most common stressors have largely not changed over time and are mostly connected to socio-economic issues, the workplace, and family responsibilities [5]. However, the COVID-19 pandemic and the consequent financial insecurity and unstable political climate have been connected to a current increase in stress to alarming levels [1]. At the same time, while we tend to speak about stress only in negative terms, stress can be healthy too: eustress can be necessary to increase productivity and obtain optimal performance [6,7].

As a response to these issues, in recent years, new technological and methodological solutions have been proposed for stress research. For instance, wearable devices that can be worn directly on the body and collect large volumes of data on physiological parameters are envisioned as potential game changers in the field. The ability to constantly measure physiological parameters, the perceived quality of measurement, and their individual and personal level frame wearable technology as a possibly crucial step in the direction of a more accurate and objective definition and measurement of stress for clinical, research, and personal purposes. More generally, the increasing adoption and development of wearable technology for health contexts is seen as a promising step in the direction of digital, personalized, and preventive medicine [8]. Furthermore, a moral hypothesis seems to guide these technological and scientific changes: the use of wearables for stress measurement is seen as a step towards a more ethical way of studying and treating stress. The use of wearables for continuous stress measurement can enable the study of chronic and long-term stress, thus opening up new ways for individuals to monitor their own stress levels and for patients to gain more awareness of their conditions [9]—a potentially promising direction for ethical principles of beneficence and autonomy. In addition, the possibility of using wearables for stress assessment in real-life settings allows for less invasive, more comfortable, and more accessible stress measurement for the general population—a promising step in the direction of ethical principles of justice. Wearable devices for the measurement of chronic stress can also be a way of enabling stress measurement for patients who would otherwise have little awareness of potentially harmful conditions [10]—again, a promising direction for principles of beneficence and justice.

Wearables, thus, seem to emerge as a more ethical way of providing biomedical research and clinical care for stress. And yet, at the same time as these promises, the more general use of wearables for health has also raised several technical, methodological, epistemic, and ethical challenges, which can cast shadows on their use for stress [11,12]. We, thus, see a crucial need to discuss the hypothesis that wearables for stress are ethically beneficial to identify real opportunities as well as risks. This is why in this paper we target the ethics of wearables for stress by applying an ethical framework based on the normative principles of bioethics: beneficence, non-maleficence, autonomy, and justice. These principles are the core of contemporary biomedical and clinical bioethics and should, thus, form the minimal basis of the application of wearable technology to stress level assessment. With this methodological approach and target, we show that wearables for stress can increase autonomy and justice by creating more knowledge of stress for more individuals; yet, issues of quality and inclusion can also negatively impact principles of non-maleficence and beneficence. This leads us to innovative results that fill in the gap of current discussions on wearable technology and stress, where ethical reflections have only partially been developed and have mostly focused on issues of data privacy and third-party misuse and, methodologically, are grounded on the interdisciplinary expertise of our group, which comprises experts in biomedical technology and stress, as well as philosophy and ethics. With this target and methodological approach, the ethical work of this paper has the expected impact of raising awareness of under-researched issues in technological development and proposing concrete solutions and recommendations, thus promoting an ethically grounded approach to wearable technology.

This paper is structured as follows. We start by presenting existing methods and limitations in the measurement and study of stress, showing that wearables are a specific way of measuring stress, which builds on existing solutions whilst promising to solve some of their limitations (Sect. Stress Level Assessment: Methods, Limitations, and Wearable Technologies). We, thus, discuss the ethics of wearables for stress by applying ethical principles from bioethics (beneficence, non-maleficence, autonomy, justice), which allows us to specify our hypothesis by identifying ethical benefits as well as challenges in this context (Sect. Applying Bioethical Principles to the Use of Wearables for Stress Level Assessment). On this basis, we show a more balanced picture of the hypothesis on the ethics of wearables for health, presenting recommendations and indications needed for the realization of this hypothesis with a focus on certification, accessibility, and inclusion (Sect. Challenges and Indications for Ethically Grounded Wearables for Stress). As such, this article is a contribution to an ethically grounded development and application of wearables for stress.

## 2. Stress Level Assessment: Methods, Limitations, and Wearable Technologies

How is stress measured in current biomedical research and care? In this section, we give an overview of current methodological approaches and their limitations as a way to contextualize and present the contribution of wearable technology in this context. Stress is a multi-faceted and expansive notion and phenomenon, which applies to several domains beyond the medical context. In this section and throughout the paper we focus on stress in mostly biomedical terms, to specify our analysis.

The foundation of stress research is based on the concepts of homeostasis and the fight-or-flight response. In the second half of the 20th century, Hans Selye was the first to define stress from a biological point of view as “a nonspecific response of the body to any demand made upon it” [13]. The response to stressful stimuli is elaborated and triggered by the stress system, integrating several brain structures to detect and interpret events as real or potential stressors [14,15]. This perception leads to the release of mediating molecules that interact with corresponding receptors in the brain and in the periphery, thus resulting in the stress response. Through physiological and behavioral mechanisms, homeostasis is restored and adaptation promoted [14,16,17].

On the basis of this conceptual and methodological tradition, two distinct types of measurement of stress are usually performed: one focusing on the subjective perception of stress and the other on the body’s response to stress. Following the first approach, stress perception is typically evaluated using questionaries designed to allow specific stress-related considerations in different scenarios. For example, the Social Readjustment Rating Scale [18] provides stress level evaluations based on life-change events experienced by the subject in previous years. The Daily Hassles Scale [19] was instead developed to assess minor life annoyances that can occur several times a day (e.g., being stuck in traffic, family fights). Other methods, such as the Perceived Stress Scale [20], focus on how different situations affect individual feelings and stress perception. Psychometric questionaries are by definition subjective and, as such, can be influenced by measurement errors, such as response bias [21]. The second measurement approach goes in a different direction and is based on the body’s response to stress associated with biochemical markers, which are measured with more invasive methods, such as blood, saliva, or urine sampling [22]. One of the main physiological reactions to the homeostatic imbalance provoked by stress is the activation of the hypothalamic-pituitary-adrenal axis (HPA axis) and the consequent release of stress hormones. The gold-standard hormone for the HPA axis’ activity evaluation is cortisol. In addition to the circadian cycle, its secretion rapidly increases due to an acute stressor and, in normal situations, a feedback loop restores normal cortisol levels. The measurement of biochemical markers is considered a way of overcoming the main limitations of measurement approaches based on psychometric questionnaires, providing a quantitative assessment of stress levels. Yet, both approaches have a crucial limitation: they cannot be used for continuous stress monitoring in real-life activities but can only provide time-limited and specific measurements—the first when the subject answers the questionnaire and the second when the laboratory test is performed. This represents a very significant limitation for an additional distinction that is at the center of the measurement and study of stress: the difference between acute or short-term and chronic or long-term stress.

**Figure 1:**
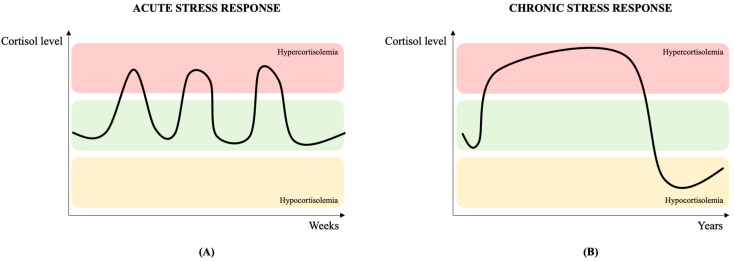
Acute (**A**) and chronic (**B**) cortisol stress response over time, changing among three different levels: normal (green), hypercortisolemia (red) and hypocortisolemia (yellow). The acute response is time-limited and, after an initial peak, cortisol returns to normal levels. In chronic stress response, the HPA axis activation is prolonged and the result is a hypercortisolemic state. Adapted from [24].

Usually, with the concept of stress, we tend to refer to acute stress (Figure 1A). This is characterized by emotional responses involving the activation of brain pathways, which support acute and time-limited adaptive processes like arousal, vigilance, and focused attention [23]. Acute stress can be characterized by negative emotional responses, such as anxiety, sadness, or anger, but can also operate as a positive mechanism of action, for example, when trying to accomplish a specific goal. Yet, if acute stress persists over an extended period of time, it can become maladaptive. Chronic stress occurs when the nervous system does not have enough time to recover from repeated acute stress events, causing negative changes in the brain areas that control sleep, immunity, and cognitive and emotional regulation (Figure 1B). For this reason, a preventive approach is essential for the timely identification and treatment of subjects at risk.

In order to measure chronic stress and consider the limitations of measurement approaches based on standard questionaries or biochemical markers, a third type of methodological approach based on physiological signals has become increasingly central in stress research [25]. Physiological signals are directly related to the body’s vital functions, such as exocrine activity, cardiac activity, brain function, and muscle excitability. The physiological signal that is most correlated with the stress response is the galvanic skin response (GSR); but a complete stress response scenario can be assessed only with a multi-parametric approach, including an electrocardiographic (ECG) signal, respiration, skin temperature, blood pressure, photoplethysmographic (PPG) signal, electroencephalographic (EEG) signal, and electromyographic (EMG) signal [21]. Physical signals can be used to measure body deformations resulting from muscle activity, including eye movements, blinks, head or whole-body movements and position, facial expressions, and voice [21].

**Figure 2:**
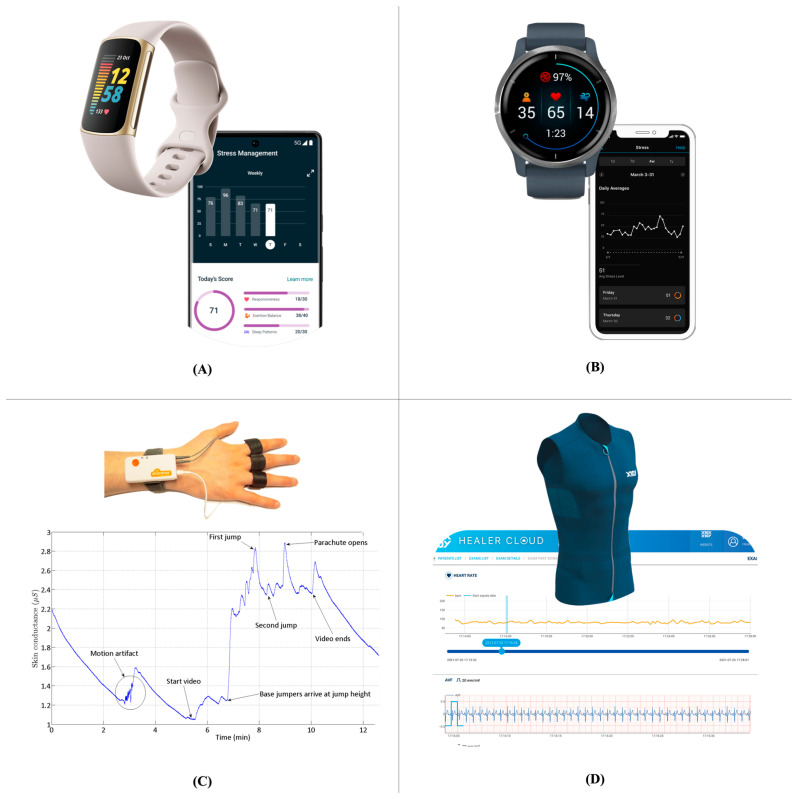
(**A**) Fitbit Change 5 wristband; (**B**) Garmin Venu 2 smart watch; (**C**) Shimmer3 GSR+ unit; (**D**) L.I.F.E. Italia Healer R2 smart garment.

The use of wearable devices for stress level assessment is an additional step in the direction of this new focus on physiological signals [26]. Wearables are devices that can be worn directly on the body and collect large volumes of data on different types of biomedical metrics and physiological signals. Wearables include smart watches, rings, garments, and bands, which can constantly measure signals, such as heart rate, physical activity, oxygen saturation, glucose levels, etc. The use of these signals for stress assessment is promising and is attracting increasing interest [10]. For instance, well-known commercial devices, such as Fitbit wristbands [27] or Garmin smart watches [28], provide a qualitative stress score based on GSR or HRV parameters. The fact that wearables are usually very comfortable and can be worn in similar ways to standard and non-monitoring devices allows the measurement of stress in real-life scenarios on an unprecedented scale. In addition, several research-oriented wearable devices are currently in development and take a more specific direction, measuring stress-related parameters according to accepted standards. For example, the Shimmer3 GSR+ unit enables GSR and PPG data acquisition in stress-related research scenarios [29]. Ensuring the accuracy of the acquired data is the main goal of this type of wearable device; but these devices are often more cumbersome and difficult to wear on a constant and daily basis and their use is focused on research scenarios. A middle point between these two approaches is emerging in the context of smart garments, which might represent a good balance between comfort in real-life scenarios and the accuracy of acquired data. Smart garments are generally designed with the aim of integrating textiles with several sensors for multi-parametric data acquisition [30]. Examples of the mentioned devices are available in Figure 2.

Therefore, various types of wearable devices are currently used and developed for health-monitoring and many of these can be used for some type of stress level assessment, responding to existing limitations connected to traditional approaches to the measurement of stress.

The use of wearable devices for stress level assessment is positioned in the context of the need to take into account the peculiarities of stress with respect to traditional healthcare scenarios. Stress level assessment is characterized by the absence of a universally acceptable definition and a gold-standard method, in particular, in real-life scenarios [9]. At the same time, it is crucial to prevent chronic-related pathology insurgence with continuous monitoring in natural environments as stress level assessment is crucially dependent on the subject and context-specific variations. Wearables can help in this direction, as becomes relevant by looking at concrete cases of application.

Occupational stress, for example, is well-researched due to its impact both on the subjects’ health and on the companies’ economy [31]. Daily working challenges can be considered an example of eustress, helping in workers’ motivation and in reaching goals; yet, the constant presence of excessive workloads and workplace tribulations often leads to chronic-stress-related symptoms for the workers [32]. Companies can face significant economic losses due to the suboptimal productivity and absenteeism of stressed-out workers [33]. In this scenario, stress level assessment through wearable devices can provide a continuous and unobtrusive monitoring method in workplaces, preventing the insurgence of stress-related phenomena [33]. A significant and concrete example of the potential impact of this application is the monitoring of people working under mentally stressful situations, such as clinicians, emergency service personnel, or high-risk workers [10].

Stress is also a fundamental challenge in sports performance. During training and competitions, athletes can face a series of stressors that could lead to performance improvement or deterioration, depending on the single athlete and the external environment [34]. For example, one stressor is represented by the expectations about performance. This can be considered a goal, referring to the ambition of the athlete to achieve a certain result, but also a source of pressure, referring to the obligation to achieve the expected results. Stress in sports can be also influenced by external events, influencing athletes’ mental and physical readiness to perform [34]. In this scenario, wearable devices could help monitor physiological parameters and stress-related metrics before, during, and after the performance. The possibility of having subject-specific models providing real-time feedback about the performance or presenting a better understanding of the athlete’s readiness for each performance are just some of the key factors of this application of wearable devices [10].

## 3. Applying Bioethical Principles to the Use of Wearables for Stress Level Assessment

What should we thus make of the new developments of wearable technology for stress we have discussed? The hypothesis we want to discuss is that the use of wearable devices in these directions constitutes a more ethical way of measuring and studying stress as the basis of possible interventions. In particular, the possible positive advancements realized in the use of wearable devices for stress level assessment indicate the hypothesis that wearables provide ethical benefits from the point of view of several key principles of bioethics, which can be defined as the discipline and theory that studies the ethical implications of developments in biomedical research and practice [35]. But is this actually the case? And, in which ways are wearables a more ethical direction?

To answer these questions, in this section, we analyze the hypothesis by applying an ethical framework based on the following normative principles: beneficence, non-maleficence, autonomy, and justice. Methodologically, our analysis is grounded on the fact that these principles are the core of contemporary bioethics [36] and, as such, they should form the minimal basis of the application of wearable technology to stress level assessment—if wearables are a more ethical way of measuring and studying stress, they should at least abide by these principles. In this way, we also ground our work on the existing literature that has applied normative principles to the use of similar digital and smart technologies in the health context [37,38]. We present the results of this work by focusing on each bioethical principle specifically and then discuss broader implications in the next section.

### 3.1. Beneficence

In bioethics, the principle of beneficence is a norm based on derivative rules including the obligation of doing no harm, balancing benefits and risks, as well as maximizing benefits and minimizing harm [36]. As such, beneficence has traditionally been considered a founding value for biomedical practice, where it constitutes both a professional obligation and an overall aim of medicine. Differently from other values (such as non-maleficence, as we will see), beneficence is about positive requirements and the principle calls for not just avoiding harm, but also benefitting patients and promoting their welfare.

In the digital health context, beneficence is often associated with the development and application of innovative technology in the biomedical context. If we apply the concept to our case of wearable devices for stress, we can see that the possibility of providing accurate and validated physiological data through wearables can be a source of several benefits for individual users and patients. As we have seen, the measurement of stress is currently limited to controlled and specific areas of research—wearables can play an important role here by allowing more individuals to track their stress and collect quantitative and valuable data to assess their stress levels.

At the same time, however, the application of wearable technology to stress assessment also raises concerns from the point of view of beneficence. First, an issue we want to highlight is the need to employ devices that are certified as medical devices for the measurement and assessment of stress. While the development of some wearables is moving in this direction, currently, several devices provide an assessment of stress that is not medically certified and produce only approximate estimates, often in a scoring system. This is potentially problematic from the point of view of beneficence as it creates unclear benefits for the user and, additionally, can create doubt and lack of trust in users of wearable technology [36]. Second, a broader question is whether the quantitative measurement of stress and knowledge of stress levels is actually beneficial for the user. This is a crucial issue that is connected more generally with the increasing datafication of health and digital health as more quantitative estimates and data become available in relation to different aspects of our health and well-being; yet it is particularly evident in the case of stress. Quantitative knowledge of stress is severely lacking in the current biomedical context and wearables can help fill in this gap; yet this also raises the possibility that more knowledge of stress can create more stress and provide few benefits for users and patients.

### 3.2. Non-Maleficence

The principle of non-maleficence is about the obligation of individuals, for instance, physicians, not to harm other individuals, for instance, a patient. Non-maleficence is usually considered the value of avoiding imposing harm on others and, as such, is often contrasted with the positive connotations of beneficence [36]. Together with beneficence and the two principles we discuss below, non-maleficence is one of the main principles of bioethics, both in the context of research and care.

When we apply the philosophical work on non-maleficence to the ethics of wearables for stress, we see wearable devices for stress assessment as a type of technology that raises considerations in terms of benefits as well as risks. Consider, again, the possibility enabled by the data collected on stress through wearables. This can be considered a way of applying the principle of non-maleficence because it potentially empowers users with new data and frees them from potentially more invasive and harmful tests. At the same time, the level of personalization that is potentially enabled by the constant monitoring of stress enabled by wearables can be considered a way of preventing harm from high levels of stress.

However, the increasing application of wearables as tools for personal stress monitoring raises issues as well. Consider, for instance, the concerning levels of overestimation that are present in current uses of wearables for digital health [11]. Overestimated levels of stress through wearable technology can be harmful from the point of view of non-maleficence, leading to alarms that can be unnecessarily concerning and harmful for individual users, if not to the overdiagnosis of stress-related conditions. In addition, the collection of new and large volumes of data on stress through wearable technology can be concerning if the data are not handled carefully and are used for harmful applications and misuse in terms of data privacy—for instance, to monitor stress levels in a work environment [39]. Finally, the aforementioned issue of the merits and implications of quantitative measurements of stress extends to concerns about non-maleficence.

### 3.3. Autonomy

In bioethics, the principle of autonomy is usually discussed in terms of the power and possibility for individuals to make rational and moral choices in their lives, individually and indigently directing them and, thus, autonomously deciding on their lives [36]. Respecting the principle of autonomy requires individuals, physicians in the biomedical context, to disclose medical information and treatment options that are necessary for the patient to exercise self-determination and support informed consent, truth-telling, and confidentiality. The principle of autonomy—also known as the principle of respect for autonomy—has dominated the discourse in contemporary bioethics, often becoming the central principle of several bioethical accounts. Independently of discussions on the merits of concentrating the bioethical discourse on autonomy [40], in the context of the use of wearables for stress level assessment, we can connect significant considerations on benefits and risks to autonomy.

Consider, again, the possibilities enabled by new and quantitative knowledge from constant stress monitoring and assessment using wearables. These can clearly be seen in connection with possible benefits for more conscious relations with stress and health status and more general empowerment in decisions on stress status, which, in turn, can be ways of promoting more autonomy in the choices of individuals affected by stress.

At the same time, however, several critical analyses of digital health have highlighted that increasing the datafication of health and monitoring can also be concerning from the point of view of autonomy [38,41,42]. When it comes to the case of stress management with wearables, the constant collection of stress data can be a source of anxiety and additional stress and be seen as an intrusion of the personal privacy that lies at the foundation of autonomy in the biomedical context. Additionally, the ability of wearable devices to detect and predict intimate health states, such as stress, can be an obstruction to the freedom and independence of autonomy and self-determination in health choices. Detection, alarms, and predictions on stress levels can direct choices and behaviors in ways that limit the autonomy of individuals, especially if devices present their inferences and conclusions on stress in ways that make them look strongly objective and high-quality.

### 3.4. Justice

Justice is generally interpreted as the principle of respecting and promoting fair, equitable, and appropriate treatment of persons. In the biomedical and health context, justice can refer to the fair, equitable, and appropriate distribution of healthcare resources determined by justified norms that structure the behaviors and connections of social cooperation [37]. The concept of justice is the focus of expansive debates in philosophy and ethics, especially in the context of biomedical research and clinical practice. For example, in the biomedical context, justice is often connected to concepts such as fairness and equity. Equity is a principle that concerns the possibility of different individuals accessing services and care in their interactions with health systems while fairness is a close principle that looks at the treatment of individuals when they interact with health systems and is implemented to make sure that they are not mistreated because of bias, discrimination, lack of consideration. Both principles are, in turn, close to justice, which is seen as the principle of promoting fair, equitable, and appropriate treatment.

If we apply this philosophical work to our hypothesis on the ethics of wearables, we can identify the following benefits and risks. Consider again the possibilities provided by an increasing data collection and monitoring generation through wearable devices—the datafication and the generation of new knowledge on stress can be seen as a benefit for justice, for instance, in the sense that it provides more people with an assessment of their stress levels during their daily life activities. This point is perhaps particularly evident when wearables are used in a specific direction: wearables are often presented as ways of tapping into new and previously unconsidered needs of individuals, thus involving more and different people in biomedical research [43]. Yet, we also want to underline that the possibilities of actually benefitting from the use of wearables in this direction can be severely hindered by current features of wearable technology. The requirement for high-quality wearable devices for stress level assessment is a welcome addition to digital health but can create more barriers in relation to the high cost of these devices. This, in turn, raises concerns for equity and, thus, justice in the implementation of wearable technology for digital health.

## 4. Challenges and Indications for Ethically Grounded Wearables for Stress

So far, in the article, we have used theories and approaches from bioethics to discuss the hypothesis according to which wearables for stress provide a more ethical way of measuring and studying stress. The results of our discussion provide a more grounded and balanced version of this hypothesis: wearables can be significant ways of applying and extending ethical principles in biomedical research on stress but can also present and create challenges for specific principles (see Table 1). Therefore, the feasibility of the ethical benefits of wearables for stress rests upon the mitigation of the issues that arise from our analysis. In other words, as a result of our analysis, it seems too simplistic to say that wearables are automatically a more ethical direction for stress research—they can be, but whether they are depends on the attention provided to new ethical issues that can emerge and existing issues that are not automatically erased by the implementation of this technology. This is also evident as a result of a possible limitation of our analysis, which has been based on the basic ethical principles of bioethics only. We have framed this choice as a basic requirement for the ethics of wearables for stressand we see this as a starting point for the use of broader ethical frameworks and discussion of other principles.

In this direction, in this section, we discuss common themes and issues that arise from our work in the paper and present recommendations and indications towards ethically grounded wearables for stress. While several of these considerations raise broader ethical concerns regarding the use of wearable technology, we will see that they gain distinct implications and raise new and different concerns in the case of stress.

### 4.1. Certification and Validation

A first common set of concerns that emerges from our analysis in the paper is connected to issues of the certification and validation of wearables for stress, particularly at the level of the data collected by these devices. As we have seen, in most cases, the use of wearables for stress measurement has not been certified by dedicated institutions and agencies and certification is often difficult to provide considering the fast technology development and marketing of wearable technology. Yet issues including the quality and accuracy, overestimation, and privacy and security of wearable data practices should be more at the center of technology development and certification considering the ethical implications we have seen throughout the paper. For subject-specific types of measurements, such as stress, the reliability of the acquired data and precision thresholds are crucial. Wearable devices can be used to collect high-grade physiological signals using gold-standard acquisition methods [44]; yet, in the wide and variable scenario of the wearable market, only a few devices seem able to collect the desired physiological measurement according to the reference standards. For example, the accuracy of heart-related parameters increases if extracted directly for an ECG signal acquired with electrodes in standard positions and not derived from optical sensors, such as the ones integrated by common wristbands. We argue that addressing these challenges requires an ethics-by-design approach right from the conceptualization of a new wearable device [45]. Starting with the selection of the best physiological signals to be incorporated into the device, both the chosen acquisition method and the subsequent data processing must align with existing reference standards. Where applicable, a comparison against established gold standards within the field should be conducted to validate the results.

For the purpose of detecting changes in physiological signals related to acute stress episodes and monitoring the insurgence of chronic events, the continuous collection of data in real-life scenarios is necessary and the absence of clear actions from the user is equally crucial. The problem, however, is that the majority of wearable devices are designed for spot measurements, only when manually activated by the user in no-movement conditions. Once again, this issue should be taken into consideration right from the initial stages of the wearable device project. Among various considerations, selecting the appropriate technological solution that facilitates continuous data acquisition is fundamental to fulfilling the crucial requirement of gathering data continuously.

Real-life monitoring also requires ergonomic solutions to be worn without interfering with activities, sleep, and movements. This, and the former, are specific issues of stress measurement: in particular, for stress-related parameters, the comfort of the acquisition device is a key factor that does not modify the integrity of the acquired physiological signals. These issues are not necessarily present or the same when wearables are used for other measurement activities, such as step counting, for example. However, they play a central role in promoting a more ethically grounded use of wearables for stress. It is, thus, imperative to acknowledge this aspect during the product development phase by carefully weighing the trade-off between user comfort and the device’s ability to accurately capture physiological data under real-life conditions.

These key challenges, thus, remain open for the use of wearable devices in healthcare monitoring applications. In this sense, we see the development of certification and policy strategies that look more actively at the ways in which wearables are used in concrete and daily contexts, rather than only at the clinical and research context, as a possible step in the direction of attending to these issues and, thus, developing ethically grounded wearables for stress [46].

### 4.2. Knowledge and Harm

A second, common concern that we have raised in our analysis is related to the possible and perhaps unexpected consequences of the knowledge about stress developed on the basis of wearable devices. As we have seen, the specific features of knowledge that can be produced through wearables—for instance, its quantitative features—and its ready and constant presence for individual users—for example, through the constant use of scoring and dedicated smart phone apps—can also be a source of harms, stress, and anxiety for users and patients [47].

On top of previous considerations on the reliability and quality of wearable stress data, we see these as concerning and somewhat paradoxical consequences of the use of wearables for stress measurement—wearables are supposed to open up stress measurements but their constant and possibly low-quality features can be harmful to stress itself, as well as the privacy and autonomy of health choices. Approaches that go in a more ethically grounded direction include the need for more transparency and accessibility of the ways in which, e.g., stress scores, are processed and presented to individual users.

When creating a device with these objectives, therefore, we argue that it is crucial to comprehensively grasp and validate the intended user demographic, ensuring that biases are minimized to prevent inaccurate conclusions. Moreover, adhering to the principle of autonomy requires a thorough investigation into how information should be conveyed to end-users and determining the primary user—whether it is intended for medical professionals or directly for the patient. This underscores the significance of a robust partnership between developers and UX/UI designers to effectively communicate information in an accurate and easily understandable manner, thereby sidestepping potential misconceptions and unwarranted alarms.

Another pivotal aspect in this direction pertains to the concept of privacy-by-design, which complements the previously discussed ethics-by-design approach [48]. When dealing with such sensitive data related to intimate states, such as stress, it becomes fundamental to ensure that all components adhere rigorously to data privacy and security regulations. Right from the development stage, assuring data anonymization and minimization becomes imperative. Furthermore, when data are shared through cloud platforms, it is necessary to implement comprehensive protective measures. This encompasses encryption, secure authentication, and robust access controls to prevent unauthorized breaches and unauthorized access. This comprehensive approach not only aligns with ethical considerations but also underscores a dedicated commitment to safeguarding user privacy while advancing stress-monitoring technologies.

These are general concerns regarding wearable technology, which become particularly significant in the context of stress. The knowledge of the individual stress levels and their potential implications can be empowering but can also be detrimental to the same condition. More awareness and understanding of these aspects can help individual users in understanding the importance of accurate measurements and the extent to which these measurements can be used and trusted when making health-related choices.

### 4.3. Accessibility and Availability

A final point that we want to discuss, as an emerging cluster of concerns in the ethics of wearables for stress, concerns the contrast between the need to raise the quality of measurements in wearable devices and their overall accessibility to the general population. Although wearables are increasingly present and actively used in the general population for a variety of different purposes, cost and accessibility remain barriers to entry for the technology [49]. In particular, in spite of large numbers of wearable users, this technology is not really inclusive of significant portions of the population—for instance, the elderly and those of a lower socioeconomic status [12]. This disparity underscores the importance of making conscious efforts toward a cost-effective design structure and optimization during development. Here, we argue that technology development should aim to introduce a solution that is genuinely valuable without being overly complex or financially unsustainable. By consciously designing for affordability and sustainability, wearable stress-monitoring technology can become more inclusive and have a positive impact on a wider range of users, ensuring that the benefits of such innovation are accessible to all.

Current campaigns to increase the general availability of wearable technology in the community and common services can lead the way for a more inclusive and diverse user basis [50]; a similar approach that is specific to the stress context could be a step towards a more ethically grounded direction for wearable technology. These concerns are common to various areas of application of wearable technology, but again, they have specific meanings in the case of stress, where goals of precision and accuracy can come at significant expenses in terms of affordability and accessibility.

## 5. Conclusions

New technologies are constantly and increasingly entering the medical field and this often comes with the idea that new technologies are more ethical ways of conducting biomedical research and delivering healthcare. This can often be the case and, indeed, the progress of the medical field is historically connected to the development of new technologies for research and care. However, as new and digital technologies enter biomedical research and healthcare, we need to carefully analyze and critically assess if they are really progressive, for whom, and which challenges are involved in these processes.

In this paper, we have followed this direction by assessing the hypothesis that digital and advanced technologies that can be directly and constantly worn on our bodies—wearables—can be beneficial for several principles of bioethics. Informed by current work on the ethics and epistemology of digital health and the bioethical literature on digital health, we have analyzed this hypothesis by applying foundational principles of bioethics to wearables for stress. While there are clear benefits in the use of wearables in this context, from the point of view of these foundational principles, we have also identified and discussed significant challenges that need our attention. As a result, our analysis presents new ethical work on advanced and upcoming technologies and leads to a more balanced and grounded picture of the role of digital health tools in concrete research and care practices. In conclusion, we want to stress that this is the starting point of work on these issues as bioethical principles are a minimal requirement for wearables for stress and more work needs to be conducted in building an expanded, ethically grounded approach in this context.

## Figures and Tables

**Table 1:** Benefits and challenges in the application of wearable technologies for stress level estimation.

	BENEFITS	CHALLENGES
**BENEFICENCE**	Expansion of new areas and inclusion of new individuals in stress measurement Quantitative physiological measurements	Necessity of validated devices Knowledge of stress can create more stress
**NON-MALEFICENCE**	Less invasive and harmful tests Feedback personalization thanks to constant monitoring	Overestimations of stress levels Need for careful handling of large volumes of data
**AUTONOMY**	More conscious relation with stress thanks to quantitative data Empowerment in decisions on stress status	Intrusion in personal privacy Obstruction of independence with personalized predictions
**JUSTICE**	Inclusion of more individuals in stress level information	Inequity of and barriers to high cost

## Data Availability

Data sharing not applicable.
